# Impact of the French National Lockdown on Admissions to 14 Pediatric Intensive Care Units During the 2020 COVID-19 Pandemic–A Retrospective Multicenter Study

**DOI:** 10.3389/fped.2021.764583

**Published:** 2021-12-10

**Authors:** Sophie Breinig, Guillaume Mortamet, David Brossier, Romain Amadieu, Isabelle Claudet, Etienne Javouhey, François Angoulvant, Catherine Arnaud

**Affiliations:** ^1^Neonatal and Pediatric Intensive Care Unit, Children's Hospital, Toulouse, France; ^2^Center for Epidemiology and Research in Population Health (CERPOP), UMR1295, Toulouse University, INSERM, Toulouse, France; ^3^Pediatric Intensive Care Unit, Grenoble-Alpes University Hospital, Grenoble, France; ^4^Pediatric Intensive Care Unit, CHU de Caen, Caen, France; ^5^Université Caen Normandie, School of Medicine, Caen, France; ^6^Université Caen Normandie, GREYC, Caen, France; ^7^Pediatric Emergency Department, Children's Hospital, Toulouse, France; ^8^Pediatric Intensive Care Unit, Hospices Civils de Lyon, Bron, France; ^9^Assistance Publique-Hôpitaux de Paris, Department of General Pediatrics, Pediatric Infectious Disease and Internal Medicine, Robert Debré University Hospital, Université de Paris, Paris, France; ^10^INSERM, Centre de Recherche des Cordeliers, UMRS 1138, Sorbonne Université, Université de Paris, Paris, France; ^11^Clinical Epidemiology Unit, University Hospital Toulouse, Toulouse, France

**Keywords:** lockdown, children, pediatric intensive care unit, viral respiratory infections, COVID 19, SARS-CoV-2

## Abstract

**Background:** After the COVID-19 pandemic reached France in January 2020, a national lockdown including school closures was officially imposed from March 17, 2020, to May 10, 2020. Pediatric intensive care units (PICUs) admit critically ill infants, children and teenagers with severe acute conditions, in particular infectious and traumatic diseases. We hypothesized that PICU admissions would be considerably modified by the lockdown.

**Aims:** The objectives of the study were to describe the type of admissions to French PICUs and to compare the occupation of PICU beds according to local epidemic conditions during the French national lockdown period, compared with the same period the previous year.

**Methods:** We conducted a retrospective multicenter study in 14 French PICUs. All children aged from 7 days to 18 years admitted to one of the 14 participating PICUs over two 3-month period (March 1, 2020, to May 31, 2020 and March 1, 2019, to May 31, 2019) were included. Analysis was based on data extracted from the medicalized information systems program (a national database used in all French hospitals, into which all admissions and their diagnoses are coded for the purpose of calculating hospital funding). Each main diagnosis was reclassified in 13 categories, corresponding to normal PICU admissions.

**Results:** We analyzed a total of 3,040 admissions, 1,323 during the 2020 study period and 1,717 during the same period in 2019. Total admissions decreased by 23% [incidence rate ratio (IRR) 0.77, 95% CI 0.71–0.83, *p* < 0.001], in particular for viral respiratory infections (−36%, IRR 0.64, 95% CI 0.44–0.94, *p* = 0.001). Admissions for almost all other diagnostic categories decreased, except intoxications and diabetes which increased, while admissions for cardiac and hemodynamic disorders were stable. Patient age and the sex ratio did not differ between the two periods. Median length of stay in the PICU was longer in 2020 [4 (IQR 2–9) vs. 3 (IQR 1–8) days, *p* = 0.002] in 2019. Mortality remained stable.

**Conclusions:** In this large national study, we showed a decrease in the number of PICU admissions. The most severe patients were still admitted to intensive care and overall mortality remained stable.

## Introduction

After the COVID-19 pandemic reached France in January 2020, a national lockdown including school closures was officially imposed from March 17, 2020 to May 10, 2020. This pandemic required profound changes in the organization of care for COVID-19 and non-COVID-19 patients. Some studies have shown that the pandemic had an impact on the number of admissions for common but serious conditions, such as acute myocardial infarction (1). A few years previously, a study on the impact of the public transport strike had shown a reduced incidence of bronchiolitis in the pediatric population (2). More recently, the 2020 lockdown was shown to have an impact on pediatric emergency department consultations and admissions for viral infections (3). This study reported that this unique “experimental” situation was responsible for a strong decrease in viral respiratory infections (including virus-related bronchiolitis), while the number of urinary tract infections remained stable. However, the impact of national lockdown on pediatric intensive care unit (PICU) admissions is still unknown.

PICUs normally admit critically ill infants, children and teenagers with severe acute conditions related to a wide range of diseases, including infectious diseases, severe injuries, shock and post-operative care after major surgery (4). We hypothesized that the complete population lockdown would result in a change in the epidemiology of patients admitted to French PICUs, with a decrease in common viral infections [based on the studies of Thélot et al. (2) and Angoulvant et al. (3)] and traumatic injury on the one hand, and an increase in domestic accidents and severe child abuse on the other. To the best of our knowledge, no study was conducted before the era of the COVID-19 pandemic on the impact of lockdown or a similar situation on traumatic injury, domestic accidents and child abuse, so the latter hypotheses were based only on the common sense of pediatric intensivists. Nevertheless, a recent adult study (5) tended to support our hypothesis with a significant decrease of medical, surgical and emergency department admissions during lockdown, in coherence with pediatric data (3).

The objectives of the study were to describe the epidemiology of admissions to French PICUs and to compare the occupation of PICU beds according to local epidemic conditions during the French national lockdown period (March–May 2020) in comparison with the same period the previous year.

## Methods

We conducted a retrospective multicenter study in 14 French PICUs, after they gave their agreement, representing 50% of the PICUs in the country. We examined activity data using the International Classification of Diseases, 10th revision (ICD-10) for diagnosis. Analysis was based on data extracted from the medicalized information systems program. This is a national database used in all French hospitals, into which all admissions and their diagnoses are entered and coded for the purpose of calculating hospital funding. All children aged from 7 days (to exclude specific neonatal diseases) to 18 years admitted in one of the 14 participating PICUs over a 3-month period (March 1, 2020–May 31, 2020) were included. During this exceptional period, adult patients could be allocated to PICU beds when adult ICU capacity was reached. As it was possible that such patients could modify the epidemiological profile of some PICUs depending on the intensity of regional COVID-19 prevalence, we reported their presence but comparison between the two periods studied was performed only on children <18 years old. Data were compared with the same period the previous year (March–May 2019). Information available from the national database included the number of patients admitted to PICUs, the patient's characteristics (age, gender, main diagnosis), medical or surgical profile of hospitalization, length of stay in the PICU, number of deaths, and number of SARS-CoV-2 positive patients. All files included anonymous data only and detailed medical records and hospitalization reports were not available.

Each principal diagnosis, extracted from the national medicalized information systems program, was reclassified in 13 categories, corresponding to normal PICU admissions: 1/viral respiratory infections; 2/non-viral respiratory diseases; 3/shock, cardiac arrests, cardiac surgery; 4/non-traumatic, non-infectious neurological diseases; 5/oncohematological diseases; 6/traumatic injuries; 7/digestive and hepatic diseases; 8/intoxications, domestic accidents, drownings, burns; 9/bacterial and viral infections (except viral respiratory infections); 10/diabetes; 11/nephrological diseases; 12/all surgery (principal or associated diagnosis); 13/SARS-CoV-2 infections. Child abuse was also collected using a specific code (principal or associated diagnosis), as it was often coded as secondary diagnosis (the main diagnosis being “head injury,” for example).

### Statistical Analysis

Continuous variables were expressed as mean (standard deviation, SD) or median (interquartile range, IQR) according to the variable's distribution and compared using the Student's *t*-test or the non-parametric Mann-Whitney test, as appropriate. Categorical variables were expressed as number (%) and compared using the chi-square (χ2) or Fisher's exact test, as appropriate.

The incidence rate ratios were calculated by Poisson regression for all admissions and for each main diagnostic category. Regional analyses were based on the reported ratios of COVID-19 hospital admissions per 100 000 inhabitants using the following thresholds: 30 or more hospital admissions for COVID-19 per 100 000 inhabitants for high prevalence and <30 for low prevalence (1). All statistical analyses were performed using Stata version 11.2 (StataCorp, College Station, TX). A *p*-value of <0.05 was considered as statistically significant.

### Ethics

In accordance with the French law on ethics, patients were informed that their codified data would be used in the study. Under French ethical and regulatory law (public health code), retrospective/prospective studies based on the exploitation of routine care data do not have to be submitted to an ethics committee. However, they must be declared and follow a reference methodology established by the French National Commission for Informatics and Liberties (CNIL).

The personal and medical data were collected and computer-processed to analyze the results of the research. Toulouse University Hospital signed a agreement to comply with the reference methodology MR-004 of the French National Commission for Informatics and Liberties (CNIL). The study was evaluated and validated by a data protection officer in accordance with the General Data Protection Regulation [Regulation (EU) 2016/679 of the European Parliament and of the Council of 27 April 2016]. Having met all the criteria, it was then registered in the Toulouse University Hospital register of retrospective studies (registration number: RnIPH 2020-103), in compliance with the reference methodology MR-004 (CNIL number: 2206723 v 0). This study was approved by Toulouse University Hospital which confirmed that ethical requirements were fully adhered to in the above report.

## Results

**Figure 1:**
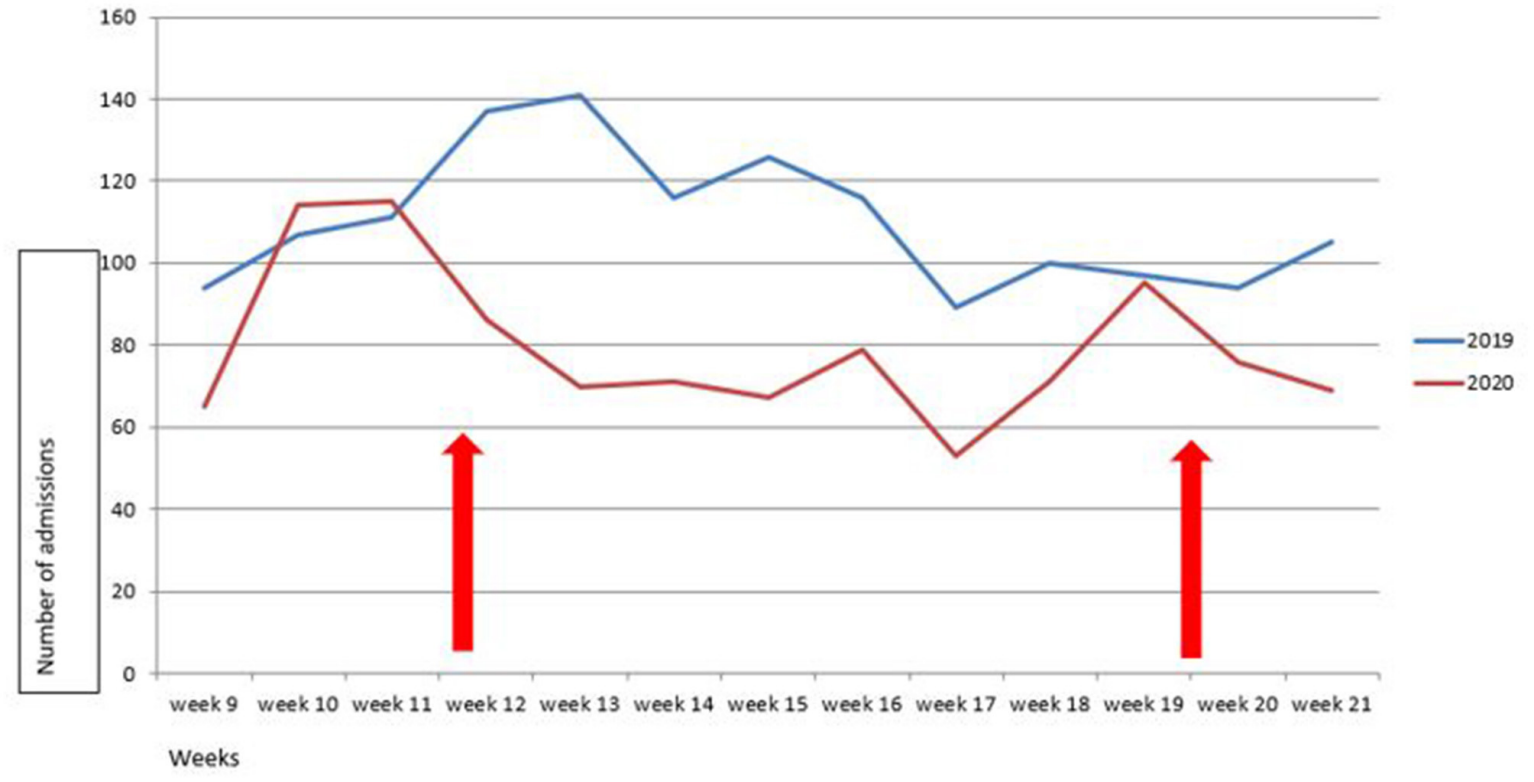
Change over time in number of weekly PICU admissions in March to May, 2019 and 2020. Lockdown was in force between week 12 to week 20 (red arrows).

**Figure 2:**
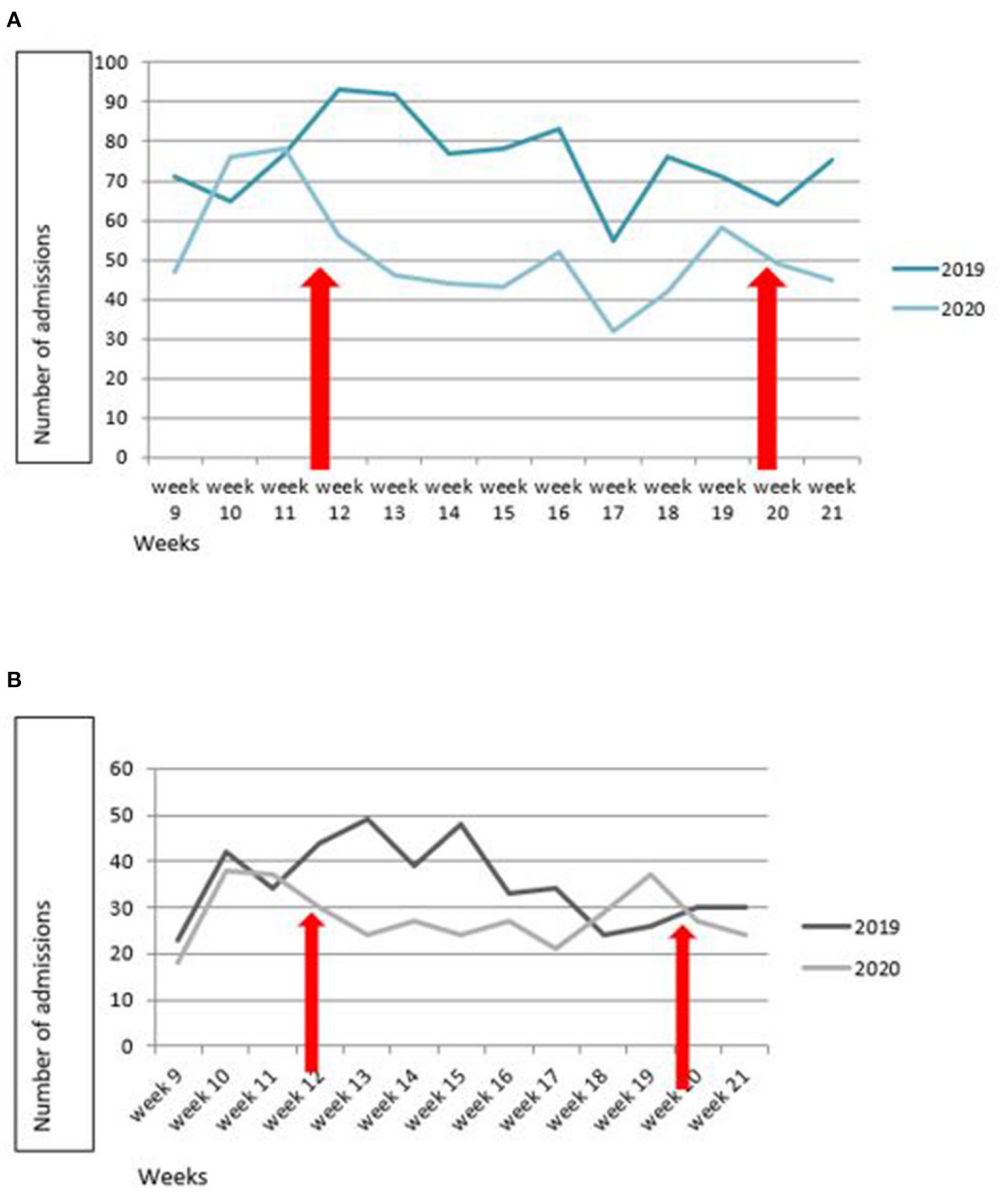
Change over time in number of weekly PICU admissions in March to May, 2019 and 2020, according to epidemic status. **(A)** Low incidence of COVID-19 hospitalization. **(B)** High incidence. Regional analyses were based on the reported ratios of COVID-19 hospital admissions per 100,000 inhabitants using the following thresholds: ≥30 hospital admissions for COVID-19 per 100,000 inhabitants for high prevalence and <30 for low prevalence, according to Mesnier et al. (1). Lockdown was in force between week 12 to week 20 (red arrows).

We analyzed a total of 3,040 admissions in the 14 participating PICUs. We compared 1,323 patients admitted from March 1 to May 31, 2020, with 1717 patients admitted in the same 3-month period in 2019. This corresponds to a 23% decrease in total admissions (incidence rate ratio IRR 0.77, 95% CI 0.71–0.83, *p* < 0.001). The weekly overall change in admissions over time is detailed in Figures 1, 2.

**Figure 3:**
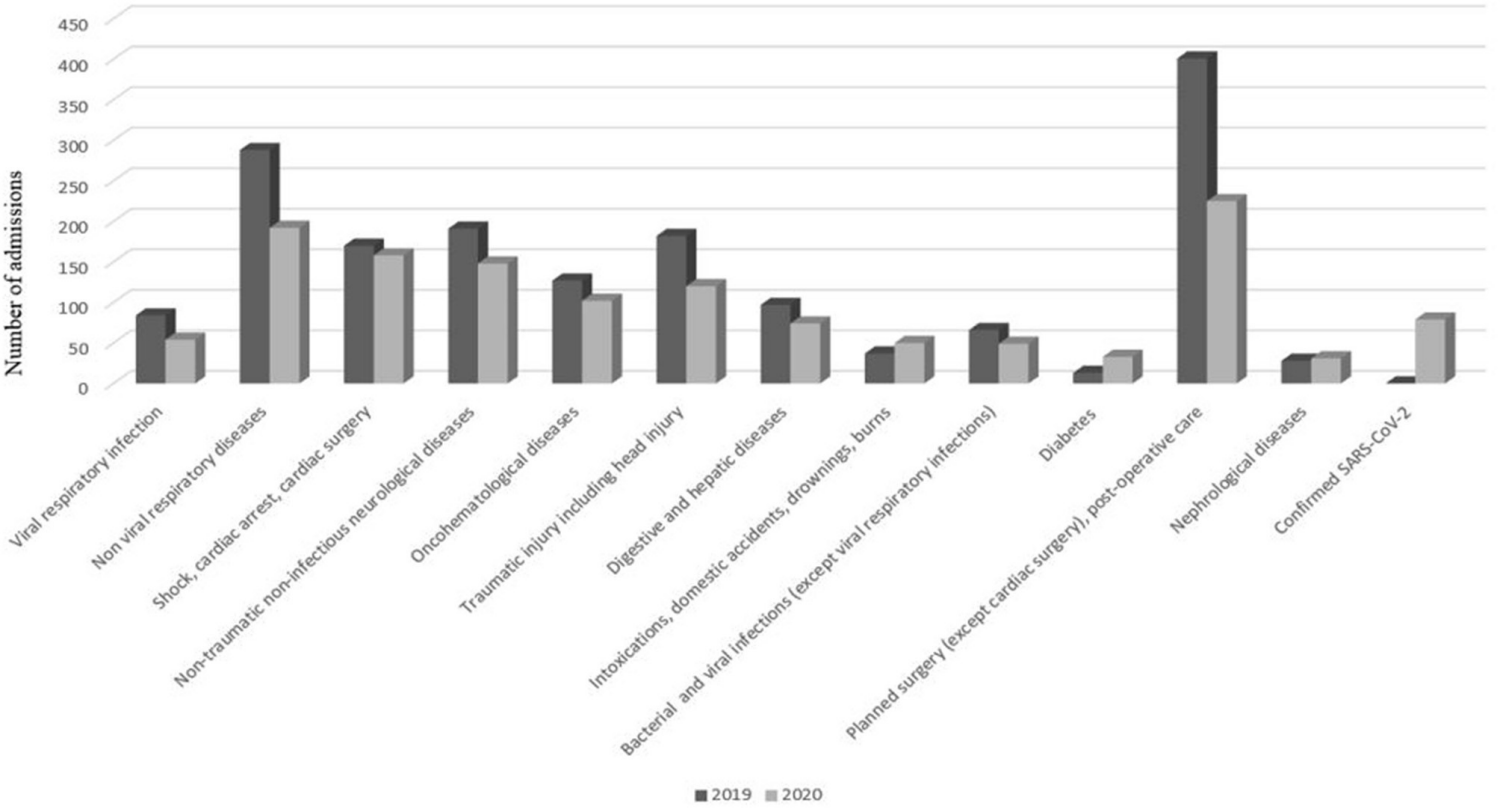
Change over time in the number of PICU admissions according to main diagnosis and to year. All admissions decreased except intoxications and diabetes.

PICU admissions decreased significantly for several diagnostic categories, in particular for viral respiratory infections (−36%, IRR 0.64, 95% CI 0.44–0.94, *p* = 0.001) (Table 1; Figure 3). On the other hand, admissions for diabetes increased significantly (+60%, IRR 2.54, 95% CI 1.30–5.25, *p* = 0.003). The incidence of intoxications and domestic accidents increased, but not significantly. Admissions for child abuse considerably decreased.

**Table 1:** Incidence rate ratio (IRR) for number of PICU admissions, all patients included and according to main diagnosis, between March–May 2019 and 2020.

	**Percentage of evolution**	**IRR**	**95% CI**	***P***
All patients	−23%	0.77	0.71; 0.83	**0.000**
Viral respiratory infections	−36%	0.64	0.44; 0.94	**0.01**
Non-viral respiratory diseases	−33%	0.66	0.52; 0.83	**0.004**
Shock. cardiac arrest. cardiac surgery	−7%	0.93	0.74; 1.16	0.51
Non traumatic non-infectious neurological diseases	−22.5%	0.77	0.62; 0.96	**0.02**
Oncohematological diseases	−20%	0.80	0.61; 1.05	0.10
Traumatic injury. including head injury	−34%	0.66	0.51; 0.83	**0.0004**
Digestive and hepatic diseases	−24%	0.76	0.55; 1.04	0.08
Intoxications. domestic accidents. Drownings, burns	**+26%**	1.35	0.86; 2.12	0.16
Bacterial and viral infections (except viral respiratory infections)	−26%	0.74	0.50; 1.09	0.11
Diabetes	**+60%**	2.54	1.30; 5.25	**0.003**
All surgery (emergency and planned)	−44%	0.57	0.51; 0.64	**0.000**
Planned surgery (except cardiac surgery), post-operative care	−44%	0.56	0.47; 0.66	**0.000**
Emergency surgery	−42%	0.56	0.47; 0.66	**0.000**
Nephrological diseases	**+10%**	1.11	0.64; 1.91	0.70
SARS-CoV-2		…	…	…
Abuse	−86%	0.14	0.02; 0.62	**0.002**
Death	**+13%**	1.14	0.80; 1.64	0.21

*Bold values in this table are those significant (p < 0.005) in the column (p) and positive percentages in the column (percentage of evolution)*.

There was no difference in age [median 2.46 (IQR 0.43; 9.49) vs. 2.32 (IQR 0.34; 10) years, *p* = 0.64] or in the sex ratio between the two periods. Median PICU length of stay was longer in 2020 than in 2019 [4 (IQR 2–9) vs. 3 (IQR 1–8) days, *p* = 0.002]. Of the 1,323 patients in the 2020 period, 69 (5.2%) were admitted for confirmed SARS-CoV-2 infection, of whom 29 (42%) were adults. The mortality rate was 5.7% in 2020 (5.3% excluding SARS-CoV-2-related deaths) and 3.6% in 2019 (*p* = 0.2). The percentage was higher because of the overall decrease in admissions, but there was no significant difference in the absolute number of deaths (61 vs. 70 deaths, + 13%, IRR 1.14, 95% CI 0.80–1.66, *p* = 0.21).

The data of all participating centers are detailed in the Supplementary Figure 1.

## Discussion

Our study revealed a significant overall decrease in PICU admissions (-23%) during the national lockdown. This decrease was observed in the majority of participating centers, including those located in a region heavily affected by the pandemic. The only exception was center 9 (Supplementary Figure 1) which admitted a large number of SARS-CoV-2 positive children due to a specific regional mode of organization.

To the best of our knowledge, this is the first multicenter study to publish relevant findings on the impact of lockdown on PICU admissions. In a recent single-center study in Maryland, USA, Graciano et al. (6) highlighted a clear decrease (−50%) in PICU admissions during lockdown. The authors postulated that this was mainly due to the significant decrease in viral respiratory diseases. We also observed a significant decrease in viral disease admissions. This decrease was confirmed by other studies performed in pediatric emergency departments (3), with a strong decrease in consultations for respiratory diseases (7–10). Our study confirmed that the same trend was also observed in more severe patients.

Our second hypothesis was that due to a decrease in car use and recreational activities, national lockdown could reduce PICU admissions for severe injury. This hypothesis was confirmed by a significant 34% decrease in trauma-related admissions, while the study by Graciano et al. (6) did not investigate this aspect. On this point, our findings are consistent with those of Hernigou et al., Keays et al. and Christey et al. concerning the epidemiology of trauma in Canada and New Zealand (11–13). These studies showed a decrease in trauma admissions during their respective lockdown periods.

Interestingly, we found that neurological diagnoses decreased significantly during national lockdown while the number of admissions for nephrological causes increased slightly. Regarding neurological conditions, our findings are consistent with those of Davisco et al. (14) in Italy, who observed a major decrease in epileptic seizures during this period. They hypothesized that this was due to an increase in parental supervision as well as a decrease in potential seizure triggers, such as viral infections.

For the other diagnostic groups in which the number of PICU admissions decreased, one may assume that there were delays in prompt diagnosis. This phenomenon has been observed in the adult population, including for acute and severe pathologies such as stroke or myocardial infarction (1). These findings led public health authorities to insist on the importance of not delaying consultation in the event of symptoms.

A rather unexpected finding was the very large increase in admissions for severe diabetic ketoacidosis. Unfortunately, the study design does not allow us to determine whether these were patients with undiagnosed diabetes or with decompensation of known diabetes, caused by the cessation of sport and/or changes in dietary habits. In children, the decompensation or discovery of diabetes often occurs in a viral or post-viral context, and one would therefore expect a decrease in this category of patients during lockdown. However, a clear opposite trend has been noted in the most recent literature. For example, Tittel et al. in Germany (15) put forward the hypothesis that great psychological stress leads to the genesis of such diabetes, although these authors showed possible variations in diabetes incidence from one year to another. Our data should therefore be interpreted with caution. Interestingly, other authors (16, 17) reported that the incidence of diabetic ketoacidosis was lower during lockdown, but that patients were in a more severe condition at admission.

It is noteworthy that our study did not reveal any significant changes in the number of admissions for hemodynamic and cardiac causes. This finding would seem to indicate that the incidence of the most severe diseases has not been affected by the current health context. This is consistent with the overall trend and increase in length of stay and mortality during lockdown.

Lastly, one of our hypotheses was that domestic accidents and child abuse would increase during lockdown. We observed a real but non-significant increase in serious domestic accidents. Other authors in a European center (18) have reported more admissions for domestic accidents without an increase in accidental intoxication, burns or inhalation/ingestion of foreign bodies. In more recent publications, serious domestic accidents requiring PICU admission were not reported.

However, due to the limited number of patients, no meaningful conclusion can be drawn from the extreme decrease in the incidence of severe child abuse. Like Caron et al. (19), we may wonder whether the incidence of child abuse may have been widely underestimated due to the absence of social contact between families. However, these authors (19) reported an 89% increase in calls to the French child protection services, in particular from neighbors.

Unfortunately, we did not study admissions for psychiatric causes. Interestingly, Mourouvaye et al. (20) reported a 50% decrease in the admission rate for suicides among French children and adolescents. This may be surprising, as it later became more evident that the pandemic was associated with high levels of post-traumatic stress (21) in healthy children, with a catastrophic impact on adolescent mental health.

Several limitations of this study should be highlighted. The retrospective design using the International Classification of Diseases for diagnosis did not allow us to provide more details about management and severity scores. Recoding by the authors of the diagnoses in 13 categories may have led to misclassification bias. There were no missing data regarding the chosen parameters, but these ones were restricted, according to the design of the study and the database type. As medical records could not be analyzed we were not able to take into account unmeasured confounding data or perform multivariate analysis. Lastly, although this was a large multicenter cohort, only half of French PICUs participated.

## Conclusion

This large multi-center retrospective study demonstrated a clear decrease in the number of PICU admissions during the COVID-19 lockdown compared to the previous year at the same time. We observed a significant decrease in viral disease. This was true regardless of the severity of the regional impact of the SARS-CoV-2 pandemic. Overall, the most severely ill patients were still admitted to intensive care and overall mortality remained stable during this period.

## Data Availability Statement

The raw data supporting the conclusions of this article will be made available by the authors, without undue reservation.

## Ethics Statement

The studies involving human participants were reviewed and approved by the reference methodology MR-004 of the French National Commission for Informatics and Liberties (CNIL). Written informed consent to participate in this study was provided by the participants' legal guardian/next of kin.

## Author Contributions

SB collected data and produced the manuscript. SB, GM, DB, EJ, and FA were involved in conception of study. RA and IC have been solicited for their expertise. CA was responsible of epidemiologic aspect and statistics. All authors contributed to the article and approved the submitted version.

## Conflict of Interest

The authors declare that the research was conducted in the absence of any commercial or financial relationships that could be construed as a potential conflict of interest.

## Publisher's Note

All claims expressed in this article are solely those of the authors and do not necessarily represent those of their affiliated organizations, or those of the publisher, the editors and the reviewers. Any product that may be evaluated in this article, or claim that may be made by its manufacturer, is not guaranteed or endorsed by the publisher.
