# Supplementary figures and images for: Impact of the French National Lockdown on Admissions to 14 Pediatric Intensive Care Units During the 2020 COVID-19 Pandemic–A Retrospective Multicenter Study
Source: Front Pediatr. 2021 Dec 10;9:764583. doi: 10.3389/fped.2021.764583 (PMC8709570; doi:10.3389/fped.2021.764583)

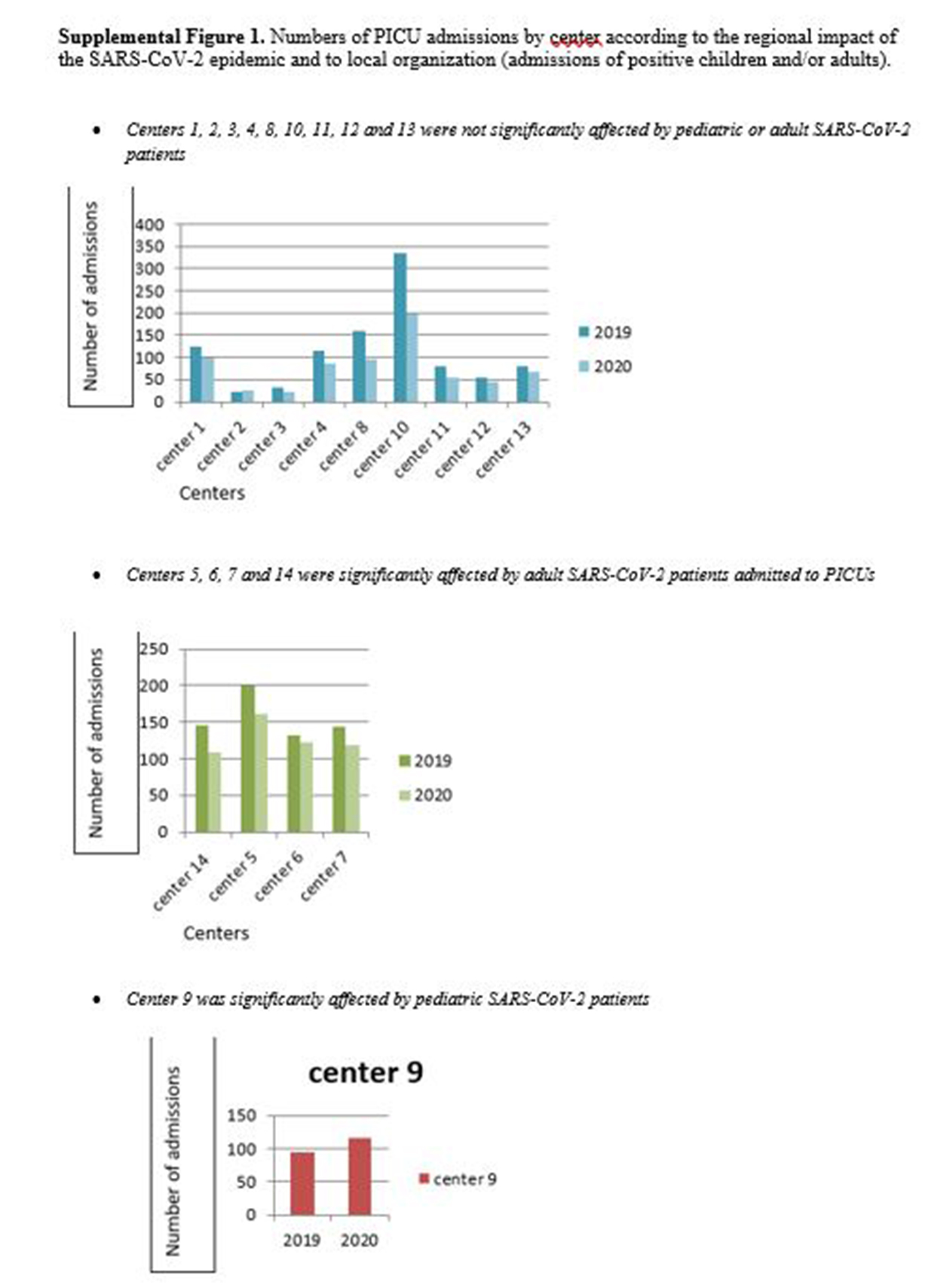

Supplement: Supplementary file 1 [file Image_1.jpg]
